# Development and implementation of an injury and illness surveillance system for team USA

**DOI:** 10.1186/s40621-024-00514-4

**Published:** 2024-07-01

**Authors:** Eric G. Post, Travis Anderson, Olivia Samson, Alexis D. Gidley, Ashley N. Triplett, Amber T. Donaldson, Jonathan T. Finnoff, William M. Adams

**Affiliations:** 1 Department of Sports Medicine, United States Olympic & Paralympic Committee, 1 Olympic Plaza, Colorado Springs, CO 80909 USA; 2 United States Coalition for the Prevention of Illness and Injury in Sport, Colorado Springs, CO USA; 3 Department of Physical Medicine and Rehabilitation, University of Colorado, Denver, CO USA; 4 University of North Carolina at Greensboro, Greensboro, NC USA; 5 School of Sport, Exercise and Health Sciences, National Centre for Sport and Exercise Medicine (NCSEM), Loughborough University, Loughborough, Leicestershire UK

**Keywords:** Elite sport, Epidemiology, Olympic, Paralympic, Methods

## Abstract

**Background:**

The purpose of this report is to provide insight and details regarding the development and implementation of an injury and illness surveillance (IIS) system for the United States Olympic and Paralympic Committee (USOPC).

**Methods:**

The development and deployment of the IIS employed a multiphase approach. First, researchers determined variables to include in the IIS using the recommendations from the 2020 IOC consensus statement for reporting sport epidemiological data. Second, the hosting and deployment platforms were comprehensively evaluated for their suitability, ease of use, flexibility, and backend data structure (for both capture and aggregation). Third, focus groups consisting of the Sports Medicine department leadership and clinicians piloted the IIS system and revisions were made based on their feedback. Pilot testing of the IIS and follow-up focus groups were then conducted among all departmental clinicians to solicit additional feedback and drive further revisions. Finally, the IIS system was piloted among providers working during the 2023 Pan American and Parapan American Games to refine the system for future Games. After reviewing all potential software platform options (electronic medical record [EMR] system, athlete management systems, secure data collection platforms), Qualtrics (Qualtrics, Provo, UT, USA) was selected to host the IIS system. This choice was made due to the inability of the EMR and athlete-management systems to make frequent updates, modify existing questions, and provide the necessary form logic for the variety of scenarios in which the IIS system would be deployed. Feedback from the department’s leadership and clinicians resulted in a number of changes, most notably being the ability to enter multiple diagnoses for a single injury event. Additionally, clinician feedback resulted in the creation of additional diagnostic codes not currently present in the OSIICS v14.0 diagnostic coding system, adding “non-sport” as an additional variable for injury setting, and developing a system for reporting return-to-sport date for time-loss injuries.

**Discussion:**

A multi-stage process of extensive planning, stakeholder feedback, and ongoing updates is required in order to successfully develop and implement an IIS system within a National Olympic and Paralynpic Committee. This process can be used to inform the development and implementation of IIS systems in other sporting organizations.

**Supplementary Information:**

The online version contains supplementary material available at 10.1186/s40621-024-00514-4.

## Background

Injury and illness surveillance systems provide valuable data that allows clinicians and researchers to understand the risk factors associated with different sports and activities, which can inform rule changes, equipment improvements, and training protocols aimed at reducing the risk of injury and illness (Bahr et al. 2020). When an athlete sustains an injury or illness it can lead to a variety of negative outcomes, ranging from missed training and competition opportunities to potential long-term health consequences (Edouard et al. 2024; Edouard et al. 2024; Clarsen et al. 2024). To address this issue, long-term goals for surveillance include establishing standardized reporting systems across sports organizations, enhancing collaboration between medical professionals and sports governing bodies, and implementing evidence-based interventions aimed at preventing injuries and illnesses, with the ultimate goal of creating a safer and healthier environment for athletes at all levels of sport (Bahr et al. 2020).

The United States Olympic and Paralympic Committee (USOPC) serves as both the National Olympic Committee and the National Paralympic Committee for the United States of America and oversees the Team USA delegations sent to represent the United States at multi-sport Games (e.g., Olympic Games, Paralympic Games, Pan American and Parapan American Games, etc.). In 2023, the Department of Sports Medicine within the USOPC completed a 5-year strategic planning exercise where a departmental purpose of delivering world-class, comprehensive healthcare to Team USA athletes was established. One of the downstream objectives of this strategic plan was to develop systems to improve operational efficiency and drive patient-centered care; the development of a robust injury and illness surveillance (IIS) system that aligns with current best practices (Bahr et al. 2020; Derman et al. 2021; Mountjoy et al. 2023; Moore et al. 2023) being one such system.

The development and implementation of the IIS fulfilled numerous needs, including a standardized method to collect injury and illness data during and between Games periods, a system that can be integrated across USOPC clinics (Colorado Springs, CO, USA; Chula Vista, CA, USA; Lake Placid, NY, USA) and the larger Olympic and Paralympic Movement within the United States (i.e., integrated into sport National Governing Bodies [NGBs]), and ensuring consistency across all providers caring for Team USA athletes. Given the importance of IIS within the scope of patient care and conducting clinical research for the prevention of illness and injury in sport, and the complexities associated with implementing such a surveillance system across a decentralized sporting environment, the purpose of this report is to provide insight and details regarding the development and implementation of the USOPC IIS system.

## Development

### History and goals

The goal of sports epidemiology is to better understand injury and illness patterns across all levels of sport in order to develop and implement effective risk reduction strategies (Bahr et al. 2020). To aid in this goal, the International Olympic Committee (IOC) published a consensus statement in 2020 to guide the standardized collection and reporting of sport epidemiology data (Bahr et al. 2020), and since that time there have been a number of supplemental publications focused on collection and reporting sport epidemiological data in specific populations or scenarios (Derman et al. 2021; Mountjoy et al. 2023; Moore et al. 2023). For example, a recent supplement outlined practices for collecting data on athlete’s mental health symptoms (Mountjoy et al. 2023). In an effort to minimize the risk of injury and illness and improve future event safety, the IOC began implementing an injury surveillance program during the 2008 Beijing Summer Olympic Games (SOG) (Junge et al. 2008, 2009), followed by an illness surveillance program in 2010 (Engebretsen et al. 2010). Since its inception in 2010, a robust multinational reporting of injuries and illnesses of athletes competing in both Summer (Engebretsen et al. 2013; Soligard et al. 2017; Derman et al. 2018, 2013, 2018) and Winter (Derman et al. 2016, 2018, 2016; Soligard et al. 2015, 2019; Nabhan et al. 2020; Valtonen et al. 2019; Steffen et al. 2022) Olympic and Paralympic Games has continued in an effort to prioritize athlete health and wellness. Through these efforts, the risk of injuries and illness occurring during the Games periods has become better quantified, which allows for more effective injury and illness prevention efforts and resource planning during large international sporting events (Engebretsen et al. 2013; Soligard et al. 2017, 2019, 2015, 2023).

The IOC and the International Paralympic Committee (IPC) conducts injury and illness surveillance during the Olympic and Paralympic Games, and the USOPC has contributed data to these efforts by utilizing the IOC or IPC Daily Medical Report (Bahr et al. 2020). However, those reports were completed on paper and provided directly to the IOC or IPC, and copies were not provided to USOPC Sports Medicine for future use. USOPC clinicians did record injury and illness information for Team USA athletes within their respective electronic medical record (EMR) system, but entry of this data was not standardized or structured in a manner to easily extract variables that were in accordance with the IOC consensus guidelines (Bahr et al. 2020). As a result, considerable personnel effort was needed to extract and clean entered EMR data into a format that could be used to deliver actionable insights to USOPC clinicians and Team USA athletes. There was a critical need for a more comprehensive and efficient system to capture and analyze injury and illness data, and thus, the Sports Medicine Research and Data and Innovation functional teams (abbreviated throughout as the IIS team) within the USOPC’s Department of Sports Medicine set out to develop an IIS system for use by USOPC Sports Medicine staff and volunteer clinicians to accomplish the following goals:

1. Provide foundational data to support USOPC Sports Medicine objectives and initiatives for improving patient health, wellness and performance;
2. Contribute injury and illness data to the IOC and IPC injury and illness surveillance program during the Summer and Winter Olympic and Paralympic Games;
3. Minimize injury and illness reporting burden for staff and volunteer clinicians while still collecting high-quality, standardized data to accomplish goals 1 and 2.

### Software platform selection

To accomplish these goals, the IIS system needed to meet several requirements. First, due to the recording and storage of protected health information within the system, the system needed to be housed in a Health Insurance Portability and Accountability Act (HIPAA) compliant platform. The system also needed a high-degree of customizability, so that questions could be continually added, modified, and formatted based both on best practices for reporting sport epidemiological data and clinician needs. Finally, the system needed to be easy to use for the clinicians in terms of account set-up and management, cost of using the system, and ease of completing an injury or illness report form.

**Fig. 1:**
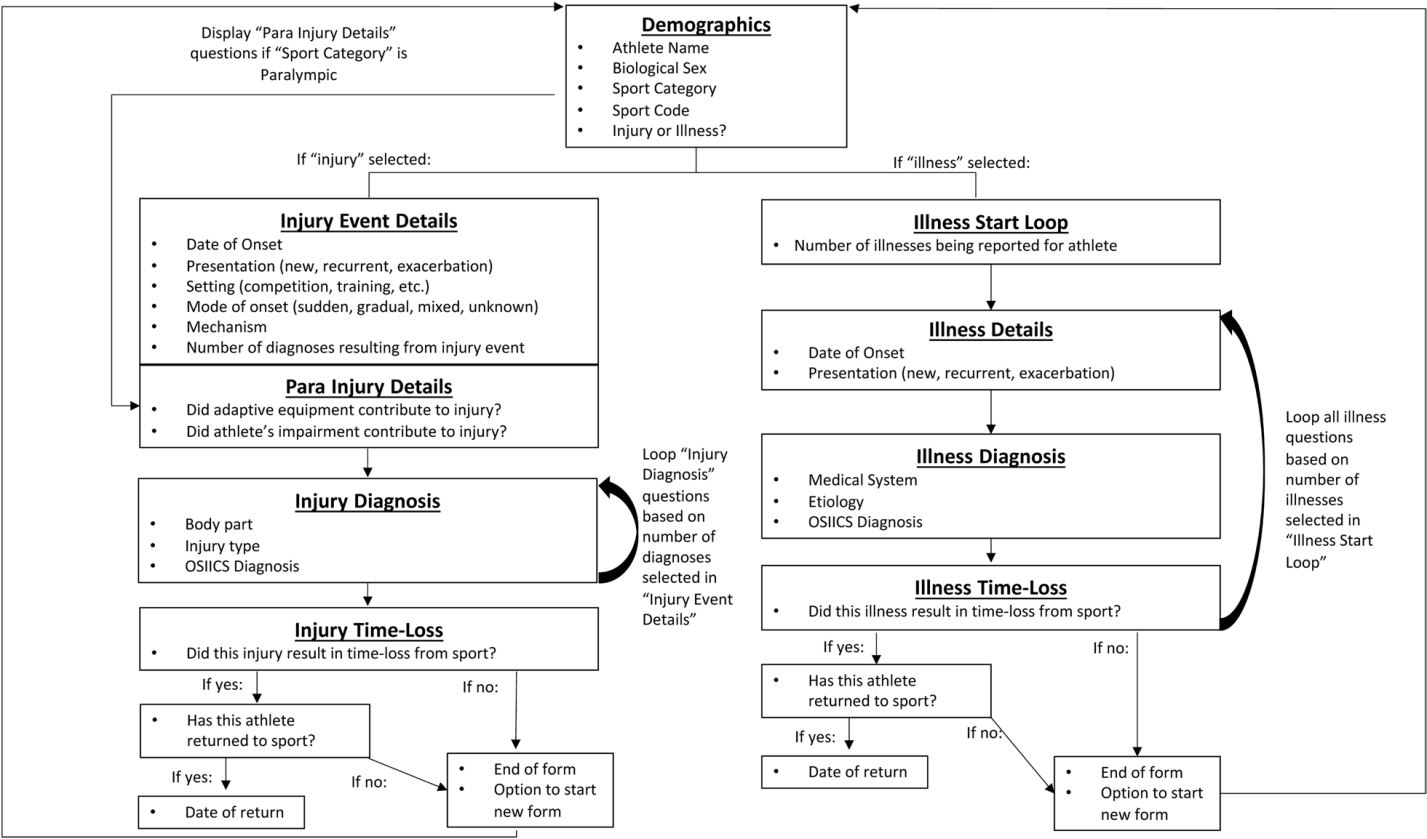
Flow diagram of branching logic required within the IIS system form

Initially, the IIS system was to be deployed through a customized form within the USOPCs EMR System (Touchworks, Altera Digital Health, Niagara Falls, NY, USA). Deployment within the EMR would have minimized repeat documentation and reduced the number of software platforms employed and staff and volunteer clinicians needed to learn and access. However, through discovery conversations with the EMR vendor, it was learned that customization of forms within the system to the degree necessary for the purpose of the IIS would be highly burdensome and would not contain functionality we deemed essential for this system. Specifically, given the large amount of branching logic needed to be included into the IIS form (Fig. 1), and with the data entry and storage within the EMR being unstructured and text-based, the extraction of data was deemed too difficult, time-consuming, and limited in capability with keeping the goal of reduced burden and increased efficiency in mind.

We also pursued implementing the IIS system within an athlete-data management system (AMS) used by the USOPC and several NGBs (Kinduct Athlete Management System, Movella, Henderson, NV); However, this system was also deemed unsuitable for the IIS system given its limited capability to create the branching logic paths for the form. Since the time of the initial discovery conversations with the USOPC’s AMS system and the writing of this manuscript, the USOPC transitioned to a new AMS vendor (Smartabase, Denver, CO, USA). Given the timing of this vendor transition and the development and IIS system discussed in this manuscript, suitability of the new AMS vendor for hosting the IIS system has yet to be fully explored.

Based on the limitations of these software platforms, we explored the feasibility of deploying the IIS within medically secure survey programs REDCap (Vanderbilt University, Nashville, TN, USA) and Qualtrics (Qualtrics, Provo, UT, USA). REDCap is a secure, web-based application for designing and implementing data collection tools and surveys, which has been used for a wide variety of patient and clinician-reported research, including in the field of injury epidemiology. However, the timeline for securing a REDCap license and establishing, building, and maintaining the organizational technical infrastructure needed to support REDCap was well beyond the implementation timeline for the USOPC IIS project. After evaluating the functionality of Qualtrics, we decided this would be an appropriate place to house the IIS system, as it met all structure, maintenance, and distribution needs..

Qualtrics is a HIPAA-compliant, web-based application that was originally designed for deploying customer experience and satisfaction surveys but has been used to implement data collection tools in a wide variety of fields, including sports medicine research. The large degree of customizability within Qualtrics allowed for the branching logic necessary within the IIS system (Fig. 1), as well as rapid iteration and revision of specific questions or aspects of the form based on clinician feedback. Furthermore, Qualtrics does not require licenses or accounts for users entering data into the platform, greatly reducing cost and logistical overhead. Conveniently, Qualtrics also has an application programming interface (API) available for automated data extraction, permitting the building of semi-automated data pipelines and workflows (see “Implementation” section). Additional benefits of using Qualtrics included the ability to input data from either a desktop/laptop computer or mobile device, the ability to require users to answer questions (to reduce missing data), and the ability to set validation requirements on certain questions (i.e., an email address must be entered in the email address field).

### Initial form development

Our definition of injury was “Tissue damage or other derangement of normal physical function due to participation in sports, that requires evaluation by a healthcare provider and results in a diagnosis.” Similarly, the operational definition of illness for the USOPC system was: “A physical health-related complaint or disorder experienced by an athlete, not related to injury, that requires evaluation by a healthcare provider and results in a diagnosis.” It is acknowledged that these definitions differ slightly from the definitions contained within the 2020 IOC Consensus statement (Bahr et al. 2020); however, the amended definitions were intended to meet the specific goals of our system to capture only injuries and illnesses that required an evaluation and diagnosis by USOPC staff or volunteer clinicians (hereafter referred to simply as ‘clinicians’) and not all health problems experienced by an athlete. As such, complaints such as general soreness that received services from clinicians (e.g., massage therapists) were deemed to be performance encounters that did not require an injury or illness diagnosis and thus not captured in the IIS system.

The choice of which variables and questions to include within the USOPC IIS reporting form was also motivated by the definitions and suggestions provided within the IOC consensus statement. For injury, these variables included date of onset, presentation, setting, mode of onset, mechanism, diagnosis, and time-loss from sport (Fig. 1). For illness, these variables included date of onset, presentation, diagnosis, and time-loss from sport (Fig. 1). Based on the sport-specific nature of the surveillance system (Bahr et al. 2020; Orchard et al. 2020), we chose to use the Orchard Sports Injury and Illness Classification System (OSIICS version 14.0) (Orchard et al. 2020) for its sport-specificity and categorical structuring rather than the International Classification of Diseases (ICD) system to simplify survey design and reduce the clinician burden. To determine injury diagnosis using OSIICS, we created a dropdown menu consisting of body parts followed by injury types that filtered down to lists ofrelevant OSIICS diagnoses that met the first two criteria. Similarly, for illness diagnosis we created a dropdown menu consisting of medical system and etiology, which then filtered down to lists of relevant OSIICS diagnoses meeting the first two criteria.

We created a demographic section that collected the athlete's name, provider email (for potential follow-up and identification of clinician, clinician credentials, clinic or setting, etc.), athlete biological sex (male, female, prefer not to say), athlete sports category (Olympic or Paralympic), and athlete sport. Based on the Para sport translation (Derman et al. 2021) of the IOC consensus statement, two questions were added specific to Paralympic athletes (1) “Did any adaptive equipment contribute to the injury?”; and (2) “Did the athlete’s impairment contribute to the injury?”. The consensus statement (Derman et al. 2021) was also used to guide the creation of several additional OSIICS codes specific to Para sport (Supplemental Online file 1).

### Initial pilot and feedback

**Fig. 2:**
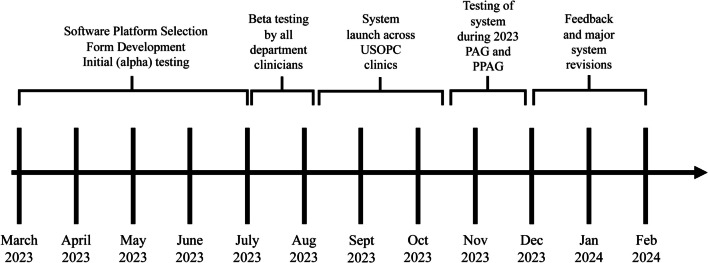
Timeline of system development

A phased approach was utilized when conducting the initial (alpha) testing of the IIS system. Departmental leadership (n = 10) and clinicians (n = 3) took part in the alpha testing round, which occurred over a 5 month period (Fig. 2). Examples of department leadership involved in the alpha testing round included the Chief Medical Officer, the Vice President of Sports Medicine, the Senior Director of Sports Medicine Clinics, as well as the Leads for each of the three USOPC Sports Medicine clinics.

An initial draft of the IIS reporting form was built within Qualtrics and then distributed to Departmental leadership for feedback. Departmental leadership members were instructed to review the form and provide feedback related to the form’s structure, clarity of wording, and additional questions/variables that should be asked/collected (Table 1).

**Table 1:** Summary of IIS revisions from alpha and beta testing, and during major system revision

Phase	Revision
Alpha testing	• Created dropdown menus to help user navigate to OSIICS diagnostic codes • Provided additional explanation of difference between “following contact with an object” and “direct contact with an object” for clarity • Re-ordered the presentation of body regions in the dropdown to be anatomical (ie. shoulder, then upper arm, then elbow, etc.) Added labels for looping to keep provider oriented when entering multiple cases (Illness 1, Illness 2, etc.)
Beta testing	• Athlete biological sex question added • Biathlon sport code updated for consistency across systems • Language for the athlete sport category question modified for clarity • Language for the sport drop-down question modified for clarity • “Outside of sport” option added for injury setting question • An additional injury type called “Impingement” created for the shoulder diagnosis drop-down menus ○ All impingement diagnosis codes (both subacromial and internal/posterior) can be found under this injury type • Three new OSIICS diagnosis codes were created and added to the illness drop-down menus for allergic reactions (see Supplemental Online file 1) Added a link on the survey end-page that redirects to a new injury/illness form
Major system revision	• Changing name field to separate “First Name” and “Last Name” fields • Changing date select tool to drop-downs • Adding question to distinguish Team USA athlete status • Sport code list updated and more comprehensive • Addition of 65 new OSIICS diagnostic codes (see Supplemental Online file 1) • Time-loss question revised for clarity

Once feedback was provided on these points, the form was revised and sent back for subsequent feedback from the departmental leadership. Following three rounds of feedback, the updated form was then distributed to three staff clinicians (two physical therapists, and one athletic trainer) for pilot testing. The clinicians were selected due to the frequency by which they conduct injury and/or illness evaluations of athletes, and thus, would be frequent users of the IIS system. Prior to the review of the IIS system, clinicians were provided an overview of the system and then were asked to pilot the system for two weeks as part of their normal patient care. Following two weeks of real-world use, the IIS team met with the clinicians to obtain feedback on the form. The feedback received, leading to further revision of the IIS system, included: (1) a more clear definition of the mode of onset, (2) adding the ability to provide additional details if the injury occurred during a competition, (3) modification of the OSIICS dropdown menus to make certain diagnoses easier to find (Table 1), and (4) the ability for clinicians to assign multiple injury diagnoses to a single injury event (Willick et al. 2021), rather than having to repeatedly entering mechanism/onset/setting information for each diagnosis or enter multiple forms when an injury event resulted in multiple diagnoses.

### Department-wide pilot and feedback

Following the alpha testing phase, the beta testing phase was initiated among all USOPC staff. The IIS system form was introduced during a USOPC staff meeting where clinicians were provided with instructions on completing the form within the IIS system. Beta testing of the IIS system within each of the three USOPC clinics occured over a one month period following the introductory presentation. Clinicians were asked to note any issues they encountered while completing the IIS system form as well as any additional feedback that was centered on improving clarity and efficiency of the form. Following the one month beta testing phase, focus groups were convened with all clinicians across the department, as well as any volunteer clinicians currently on a volunteer rotation at the time of testing, with each focus group consisting of 3–4 individuals to ensure that each clinician’s voice was heard. During the focus groups, clinicians were provided the opportunity to provide feedback, ask questions about the system, and make suggestions for improving the IIS system as a whole.

Following the focus group meetings and the feedback obtained by the IIS team during the process, another round of major revisions ensued that incorporated the feedback provided by all clinicians during the focus groups. This revision round included the creation of posters and stickers with a QR code linking directly to the IIS system form, creation of a training video and materials to be incorporated into the onboarding of USOPC Sports Medicine volunteers at all clinics (each clinic hosts volunteer clinicians for 1–2 week rotations throughout the year), modification of wording throughout several questions for clarity, adding an additional option for the injury setting question for injuries occurring outside of sport, creating a link at the end of the form that redirects to start a new form, and creation of several new OSIICS codes for allergic reactions (Table 1, Supplemental Online file 1). The link to start a new form was added to the end of the form in case clinicians were documenting multiple athlete evaluations at the same time.

At this time, members of the IIS team also became integrated into the interdisciplinary patient care meetings at each USOPC Sports Medicine clinic, which serve to provide an interdisciplinary discussion of current patient cases at each clinic. The presence of the IIS team at these meetings served two primary functions: (1) to identify injury and illness records that should appear in the IIS system but were not yet entered (i.e., identify and complete data entry), and (2) to obtain return to sport dates for athletes identified as having a time loss injury in the system who had since returned to sport. While this system worked for obtaining return to sport dates for athletes evaluated at each clinic, a separate system was created for obtaining return to sport dates for athletes evaluated during Games periods. Following each Game period, members of the USOPC Sports Medicine Athlete Healthcare team whose primary role involves directly interfacing with Team USA athletes to help them navigate the USOPC Medical Network and larger U.S. healthcare system, assisted with conducting targeted follow-up with athletes to determine return to sport status. Many other avenues were explored for collecting return to sport data, including a separate Qualtrics form, but through careful deliberation and practical experience, increasing human involvement in the data collection process became fundamental to obtaining accurate and complete data.

### Data architecture

The intent of the IIS system was to achieve the goals outlined above, all of which support the mission of providing world-class, comprehensive health care to Team USA athletes. As such, while the data collected is and will continue to be valuable to the scientific and medical communities as a whole through the publication and presentation of Team USA IIS system data, we also structured the system to be a clinician-facing tool. That is, we designed this system to permit the information to be readily and easily communicated to Team USA Sports Medicine staff to inform and improve clinical practice, via a robust data architecture and reporting system.

**Fig. 3:**
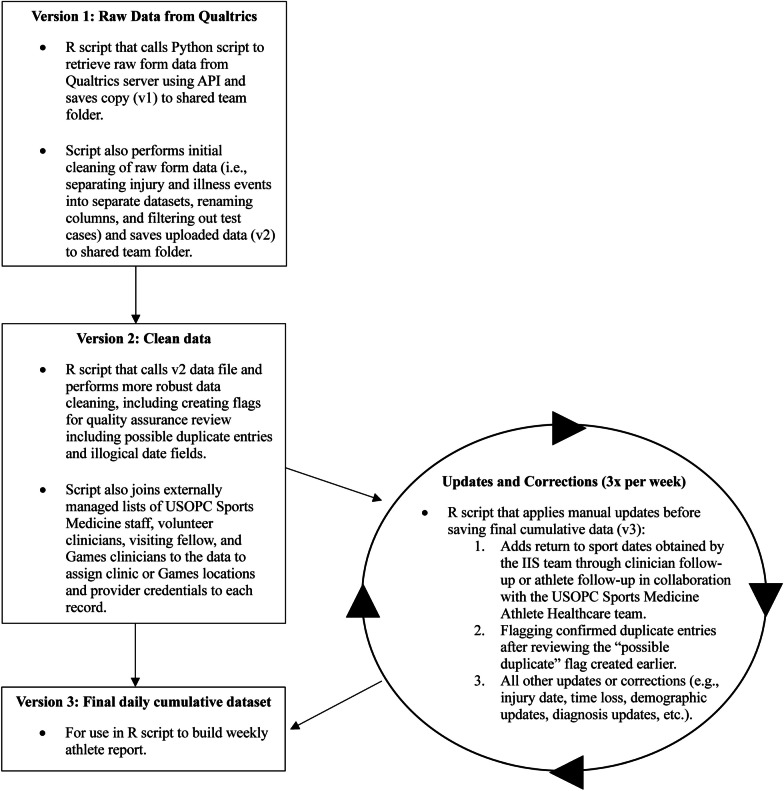
Flow chart of data management and quality assurance processes

Data management and quality assurance processes were also conducted using the programming language R. A sequence of R scripts (Fig. 3) were developed in order to organize the major steps of data processing and allow for flexible updating and revising throughout the initial implementation process. Injury and illness datasets were saved separately due to the different questions asked for injury and illness events, but all data management and quality assurance processes were conducted in parallel in the same scripts. The first two scripts in the process for data extraction and cumulative file development were scheduled to run automatically every morning to establish a regular data processing schedule in addition to processing the data occasionally for specific requests. Data was saved in folders on a shared drive accessible to members of the USOPC Department of Sports Medicine.

The first script retrieves and saves the raw form data (v1) from the Qualtrics server using a Python script, conducts initial cleaning, and saves the updated data (v2) to the shared folder. The second script performs more comprehensive data cleaning, flags potential duplicates, and integrates external lists of medical staff to assign clinic or Games locations and provider credentials to each record. A third script allowed for manual updates to the data, including return-to-sport dates and flagging duplicate entries. Possible duplicates are flagged based on a key combining athlete name and body part of the injury or medical system of the illness. If the keys from two different form entries match, all events entered in the two forms will be flagged for review. One example of duplicate entry occurring is when two clinicians see the same athlete in active care for the same injury or illness and both complete the form. Maintaining a robust duplicate flagging and review process is essential to determine an accurate number of injuries and illnesses occurring in Team USA athletes. The possible duplicate entries are output as a separate file that is reviewed after each Games period and on a monthly basis for all non-Games entries. This third script also includes all other updates or corrections (e.g., injury date, time loss, demographic updates, diagnosis updates, etc.), which are primarily obtained during IIS team engagement with clinicians, and are primarily due to changes in information on the event (i.e., injury diagnosis updated after imaging), or incorrect entry in the form by the clinician. Finally, the third script saves the final report-ready data (v3).

Additional R scripts were developed to take the cumulative file and create customized datasets output as spreadsheet files (Microsoft Excel, Microsoft Corporation, Redmond, WA, USA) to further supplement Games reporting and clinician engagement with the system. One script is the Weekly Athlete Rounds file build, which pulls all entries recorded for a given USOPC clinic location (excluding Games entries) in the past week. This file serves as a dynamic facilitation tool for weekly provider meetings, and also identifies the time loss events entered that require a return to sport date follow-up by the IIS team. The file includes a snapshot of information on each IIS entry, including athlete name, clinician name, sport, sex, diagnosis, injury or illness date, injury onset or illness presentation, and time loss. In addition to the subset of IIS data, the file includes a field for clincians to select if the record is “open” or “closed” based on case status (i.e., is the athlete still seeking care with clinicians for the injury or illness), which allows the file to be customized by clinicians and events to be carried forward or dropped from the list for the next week’s file. The second script is the Games file build, which during Games periods pulls daily Games injuries and illnesses for reference and tracking by the IIS team, and to support USOPC participation in IOC Games surveillance activities.

In addition to the embedded quality assurance checks during the data cleaning process, a final R script conducts checks for any additional review including potential human error in form entry (e.g., invalid clinician email address, impossible date relationships [e.g., injury date is a date in the future, return to sport date is before injury date]). The IIS team is continually updating and refining the quality assurance process in the early stages of the IIS system development.

### Reporting

A key component of any injury and illness surveillance system is prompt and clear reporting of data back to relevant stakeholders, so they can use that data to inform clinical decision-making or the development of preventative interventions. Within the USOPC, the Sports Medicine Research team has developed a communication and translation policy, the goal of which is to effectively disseminate knowledge gained through Sports Medicine Research studies to Team USA athletes, USOPC clinicians, internal USOPC stakeholders, NGB partners, the broader scientific and medical communities, and to the general public.

As part of this effort, the IIS team discussed multiple options for communicating IIS system data back to clinicians, including online hosting of data dashboards, oral presentations, and static PDF reports. We determined that successful communication of the IIS system data required (1) timely and consistent communication, (2) relevant data, (3) easily digestible and aesthetically pleasing delivery of information, (4) actionable insights, (5) flexible viewing methods, and (6) an automated or semi-automated and flexible creation method. While online dashboards mostly achieve these goals, legal and technical concerns for some platforms (e.g., RShiny applications hosted on shinyapps.io use Amazon Web Services and are not HIPAA compliant unless a privately managed server is engineered) eliminated those options. Other options, such as dashboards on an internally managed platform (e.g. Tableau), may have served this process; however, it was deemed that managing Tableau account dashboard access would become laborious and would require more time from clinicians to navigate to and interact with the dashboard. As such, it was determined that the most effective communication method would be a static PDF summary data report that could be emailed to USOPC Sports Medicine staff and volunteer clinicians and viewed on all electronic devices with no need to download specific applications or navigate to any URLs.

**Fig. 4:**
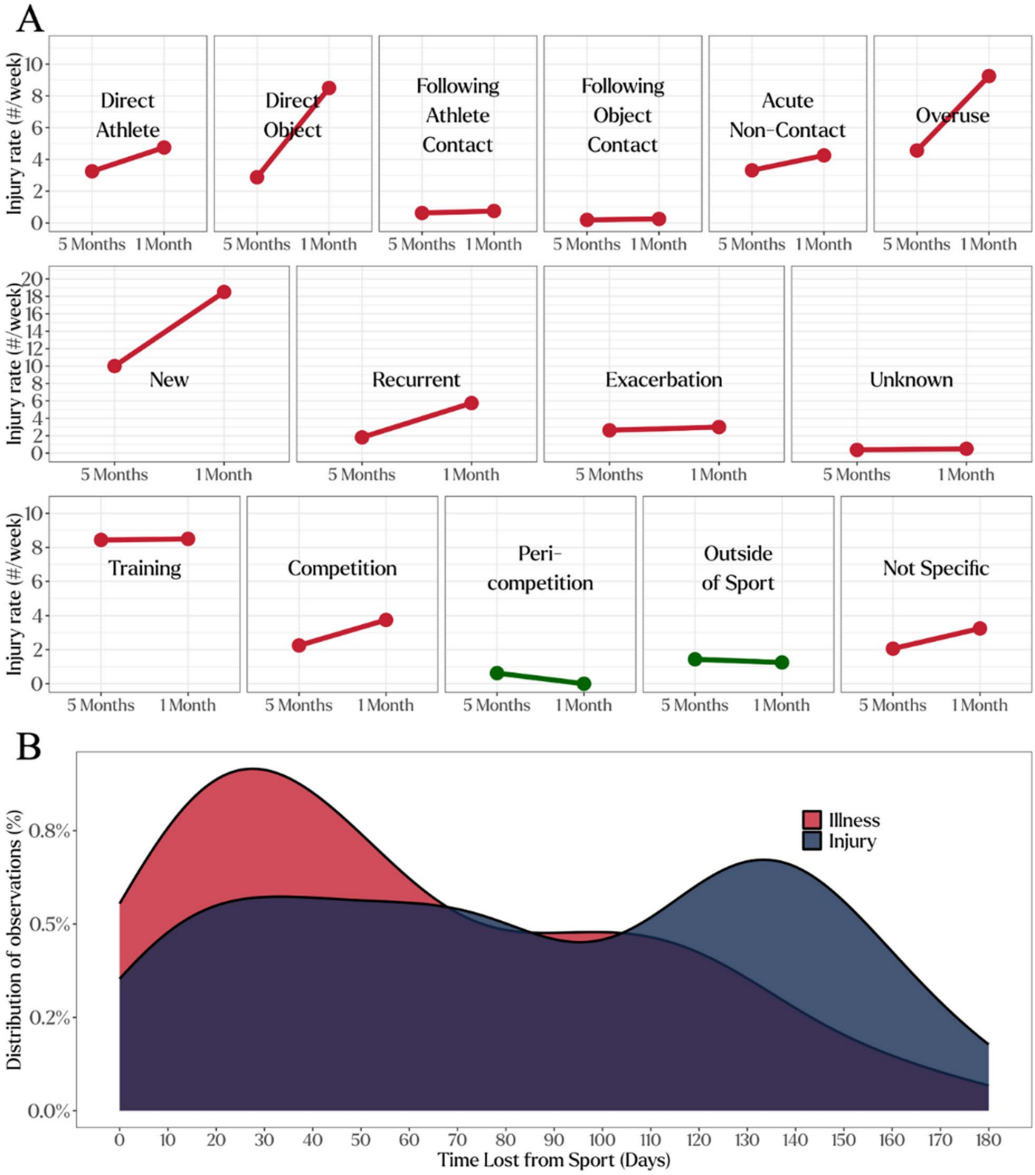
Example plots from monthly reports: **A** comparisons of injury counts per week between the previous month and the previous five months for mechanism, setting, and timing **B** time to return to sport following a time loss injury or illness

To enhance the scope of data insights we could provide to clinicians, several reports were developed and provided at varying cadences. First, we built a weekly reporting structure to be sent to clinicians every Monday that contained IIS system data from the previous week. These reports were all deliberately limited to only two pages for reporting both injury and illness data in an effort to concisely summarize the most important information from the previous week. Separate reports were generated for the entire USOPC Sports Medicine department and for each of the three Sports Medicine clinics. Next, monthly reports were created to be distributed on the first Friday of every month, which contained more elaborate data analysis and visualizations including information pertaining to the time to return to sport (Fig. 4A), and a comparison of specific injury or illness category (body part or medical system) counts between the previous months and the preceding five months (Fig. 4B). In an effort to make the data maximally actionable, the IIS team identifies three major take-home messages from the data and provided a short narrative at the beginning of each monthly report. During the report development phase, feedback was garnered from all clinicians and department leadership, and this feedback consisted of minor suggestions related to formatting, colors, and labeling of figures for clarity.

To achieve the goal of automating the report generation process, R was utilized to build the reports using the RMarkdown file format. An advantage of RMarkdown files is that they permit the use of R code (for visualizations) and the LaTex markup language for rendering and knitting the RMarkdown file to PDF reports. To aid in the automation of reports, the code for all data visualizations was written to be highly flexible and dynamic to accommodate scenarios that were anticipated to occur and re-written when unforeseen peculiarities with the data were seen. For example, all figures dynamically adjust the axis ranges in response to varying numbers of injuries and illnesses recorded for a given time period. An example of the RMarkdown code used in the reports is available in Supplemental Online file 2.

To automate the generation of the PDF reports, two steps were required. First, a Python script was triggered at a given time to retrieve the data from the Qualtrics server which is subsequentially cleaned and wrangled by separate R scripts, as outlined in the Data Architecture section above. Second, an R script was used to call the appropriate RMarkdown files and move the knitted PDF reports into a shared file directory. The reports then undergo a quality assurance process by three members of the IIS team, before being distributed to the appropriate clinicians via email. This semi-automated report generation process is highly efficient while still permitting a data review and quality assurance process and results in comprehensive, meaningful, and effective data communication. As the IIS system further develops to include individual NGB partners, future reports will include *à la carte* reports for the NGB medical leads, highlighting the information that is most critical to improving their specific clinical care scenario. These customized reports will be built and automated in a similar manner.

## Implementation

### System launch and first games period

Following the alpha and beta testing stages and resulting revisions, the USOPC IIS was officially launched for use among clinicians at the three USOPC Sports Medicine clinics in August 2023. Following the official launch of the system, the 2023 Santiago Pan American Games (PAG) and Parapan American Games (PPAG) were identified as an opportunity to implement and test the system during a Games period. Before the start of each Games period (PAG: Oct 20-Nov 5, 2023; PPAG: Nov 17–26, 2023), training materials were created and distributed as part of the onboarding for all USOPC Sports Medicine staff, NGB medical providers, and volunteer clinicians selected for those Games. This training included both group sessions as well as office hours where individuals could have questions answered and practice documenting within the IIS system.

Because this was the first implementation of the system during a Games period, a concurrent audit was conducted during each Games that compared all entries in the IIS system against documentation of injury and illness evaluations within the EMR. This audit revealed a total of 128 medical conditions (86 injuries, 42 illnesses) were evaluated during the PAG and documented in the EMR. However, 41% of these conditions (52 entries) were not documented in the IIS, and had to be entered manually after the Games by the IIS team. As a result of the large amount of missing data during PAG, several changes were made to the implementation process prior to the start of PPAG. Pre-games training materials and sessions were revised to put a greater emphasis on clearly communicating the situations when the form should be completed. Additionally, a member of the IIS team periodically joined daily clinician meetings throughout the Games period, to emphasize complete documentation within the IIS and also communicate trends in injury, illness, and other data. Consequently, out of a total of 111 medical conditions (66 injuries, 45 illnesses) evaluated during PPAG, only 14% (16 entries) were not documented within the IIS system. While the degree of missing data was improved during PPAG, consistent issues were identified in the missing data that will allow for further improvement of the system during future Games. Specifically, the missing data tended to be from evaluations of injuries or illnesses that originated prior to the Games but were exacerbated during the Games. Therefore, future training for USOPC Sports Medicine staff, NGB medical providers, and volunteer clinicians working during a Games will emphasize the need to document all injury and illness evaluations, whether they be for a new, recurrent, or exacerbated condition.

### Major system revisions

During the implementation of the system at all USOPC clinics in the fall of 2023 and during the 2023 Santiago PAG and PPAG, ongoing feedback was gathered from clinicians interfacing with the system during this time. All of the feedback collected during this time was collated by the IIS team, and common issues were identified to be addressed during a major system update in January 2024. This system update consisted of three major revisions within the system in addition to several more minor changes that primarily consisted of restructuring questions to provide cleaner data (Table 1). One example of a minor revision was splitting the “Athlete Name” field into separate “First Name” and “Last Name” fields to improve deduplication and quality assurance processes. Another minor revision was the addition of a question to clarify whether an athlete was a Team USA athlete (an athlete who has been selected to represent Team USA at a Games or at a competition on the pathway to a Games) or did not meet this qualification (ie. an athlete who came to a training center for a developmental camp and sustained an injury or illness).

The first major revision to the system was updating the sport code list used to categorize an athlete’s sport. The original sport code list in the system was found to be outdated and not comprehensive of all sports. A new list was developed to include all sports and group them by sport-discipline groups that would be efficient for clinicians to select alphabetically (e.g., “Gymnastics—Artistic”, “Gymnastics—Rhythmic”, and “Gymnastics—Trampoline”, instead of “Artistic Gymnastics”, “Rhythmic Gymnastics”, “Trampoline Gymnastics”). Similarly, while the OSIICS system is more sport-specific compared to the ICD system, it has fewer diagnostic codes. Therefore, after using the system for several months, clinicians provided many additional diagnostic codes that would be helpful to add to the OSIICS system (Supplemental Online file 1). Finally, clinicians suggested revising the question related to time-loss to reduce confusion about how to answer if the athlete initially finished the competition/training but could not participate in the future. To accomplish this, the response options for this question were split into three answers (each with discrete definitions), instead of two answers (with one answer that contained two definitions in an “OR” statement) (Table 1c).

### Future directions

The initial development and implementation of the USOPC IIS system is the first step in creating an effective injury and illness surveillance system for Team USA. Continual growth and improvement is necessary to deploy a system that meets the ever changing needs of Team USA athletes and USOPC Sports Medicine staff and volunteer clincians. While the current IIS system is capable of recording injury and illness occurrence, it does not currently include a system for tracking mental health symptoms/events, which is a key component of the care provided to Team USA athletes by the USOPC Sports Medicine Team.

Additionally, the IIS currently does not include a system for capturing athlete exposure, which is necessary for the calculation of incidence and comparisons of risk between cohorts. While we are able to capture exposure (in athlete-days) during Games periods through athlete travel and accommodation records, capturing more specific measures of athlete exposure and capturing exposure outside of Games periods is more complicated and difficult. A major next step for the USOPC IIS is developing exposure tracking tools that can be implemented alongside the injury and illness reporting form, while also ideally being tailored to specific sports. As part of this effort, a second major goal for the IIS is to partner with the various NGBs within the United States to capture foundational data on injury and illness trends across the decentralized landscape of elite sport in the United States, to ultimately support improving the health and performance of Team USA athletes.

Finally, while the initial development and implementation of the system within Qualtrics has been successful, this use case was not the original intention of that platform, and thus, this project has required extensive work to transform the platform into an IIS system. Identifying and transferring the IIS system into a software platform that is more specifically designed for medical (and ideally sport) record collection will likely streamline the process of both data collection and management.

## Conclusions

**Fig. 5:**
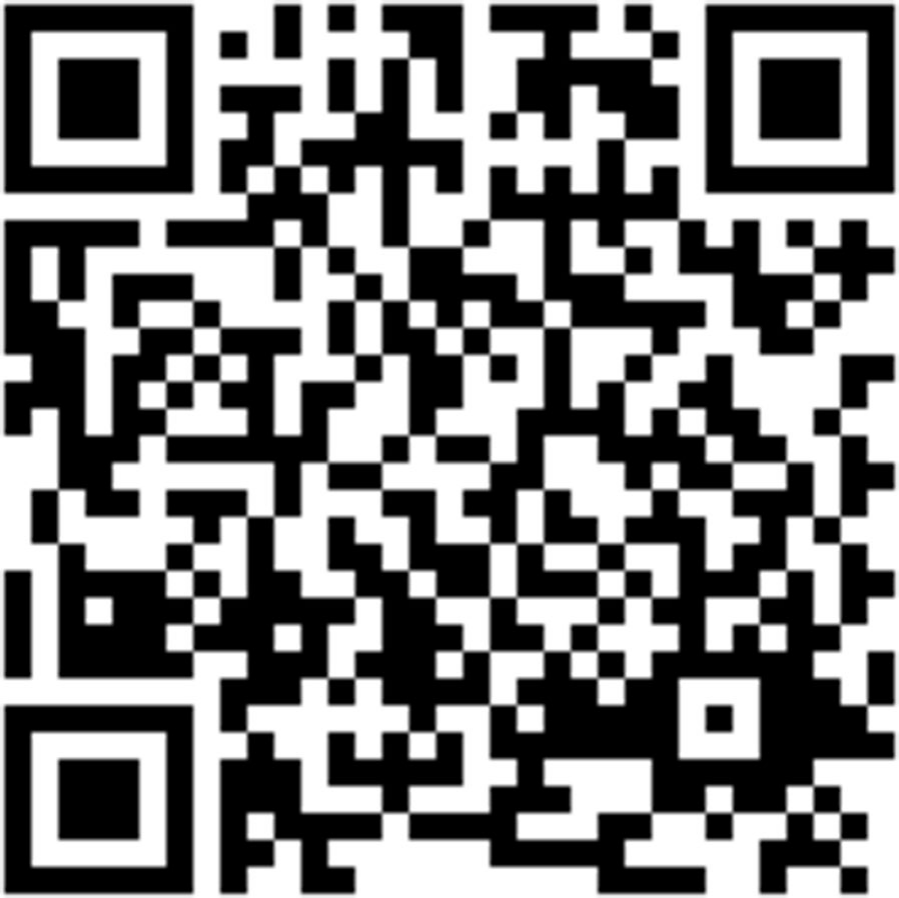
QR code linking to a non-active, but current (as of publication date) version of the USOPC IIS system form within Qualtrics

The successful development and implementation of an IIS system for the USOPC required a multi-stage process consisting of extensive planning, stakeholder feedback, and on-going updates. The goal of this report was to outline a process that can be used to inform the development and implementation of IIS systems in other sporting organizations. To support this goal, a non-active but current (as of publication date) version of the USOPC IIS system form can be accessed via the QR code in Fig. 5.

### Supplementary Information

Supplemental Online file 1. New OSIICS codes created and added during development and implementation of IIS system.

Supplemental Online file 2. Example RMarkdown code used to generate IIS reports.

## Data Availability

All data generated or analysed during this study are included in this published article [see Fig. 5 and supplementary materials].
